# Risk classification and door-to-antibiotic time in patients with suspected sepsis

**DOI:** 10.1590/1518-8345.6635.4065

**Published:** 2023-12-04

**Authors:** Ana Paula Souza Lima, Gláucio de Oliveira Nangino, Fabiana Fernandes Rego Soares, Joyce de Carvalho Xavier, Maria Cláudia Martins, Arnaldo Santos Leite

**Affiliations:** 1 Hospital da Polícia Militar de Minas Gerais, Centro de Terapia Intensiva, Belo Horizonte, MG, Brasil.; 2 Hospital da Polícia Militar de Minas Gerais, Diretoria Técnica, Belo Horizonte, MG, Brasil.; 3 Hospital da Polícia Militar de Minas Gerais, Unidade de Internação, Belo Horizonte, MG, Brasil.; 4 Hospital da Polícia Militar de Minas Gerais, Centro de Terapia Intensiva, Belo Horizonte, MG, Brasil.; 5 Universidade Federal de Minas Gerais, Faculdade de Medicina, Belo Horizonte, MG, Brasil.

**Keywords:** Sepsis, Triaje, Hospital Emergency Service, Anti-Bacterial Agents, Emergency Nursing, Critical Care, Sepsis, Triaje, Servicio de Urgencia en Hospital, Antibacterianos, Enfermería de Urgencia, Cuidados Críticos, Sepsis, Triaje, Serviço Hospitalar de Emergência, Antibacterianos, Enfermagem em Emergênci, Cuidados Críticos

## Abstract

**Objective:**

to evaluate the association between risk classification and door-to-antibiotic time in patients with suspected sepsis.

**Method:**

retrospective cohort study, with a sample of 232 patients with suspected sepsis treated at the emergency department. They were divided into 2 groups: with and without risk classification. Once the door-to-antibiotic time was identified, one-way analysis of variance was performed with Bonferroni *post hoc* test or independent Student’s t-test for continuous quantitative variables; Pearson correlation tests, point-biserial correlation or biserial correlation for association analyses; and bootstrap procedure when there was no normal distribution of variables. For data analysis, the Statistical Package for the Social Sciences software was used.

**Results:**

the door-to-antibiotic time did not differ between the group that received risk classification compared to the one that was not classified. Door-to-antibiotic time was significantly shorter in the group that received a high priority risk classification.

**Conclusion:**

there was no association between door-to-antibiotic time and whether or not the risk classification was performed, nor with hospitalization in infirmaries and intensive care units, or with the length of hospital stay. It was observed that the higher the priority, the shorter the door-to-antibiotic time.

## Abstract (es)

**Objetivo:**

evaluar la asociación entre la clasificación de riesgo y el tiempo puerta-antibiótico en pacientes con sospecha de sepsis.

**Método:**

estudio de cohorte retrospectivo, con una muestra de 232 pacientes con sospecha de sepsis atendidos en el departamento de emergencias. Se dividieron en 2 grupos: con y sin clasificación de riesgo. Una vez identificado el tiempo puerta-antibiótico, se realizó un análisis de varianza de un factor con la prueba *post hoc* de Bonferroni o la prueba t de Student independiente para variables cuantitativas continuas; pruebas de correlación de Pearson, correlación biserial puntual o correlación biserial para análisis de asociación; y procedimiento de *bootstrap* cuando no había distribución normal de variables. Para el análisis de los datos se utilizó el *software* *Statistical Package for the Social Sciences*.

**Resultados:**

el tiempo puerta-antibiótico no difirió entre el grupo que recibió clasificación de riesgo en comparación con el que no fue clasificado. El tiempo puerta-antibiótico fue significativamente más corto en el grupo que recibió una clasificación de riesgo de alta prioridad.

**Conclusión:**

no hubo asociación entre el tiempo puerta-antibiótico y si se realizó o no la clasificación de riesgo, ni con la hospitalización en enfermería y en unidad de cuidados intensivos, ni con la duración de la estancia hospitalaria. Se observó que cuanto mayor era la prioridad, más corto era el tiempo puerta-antibiótico.

## Abstract (pt)

**Objetivo:**

avaliar a associação entre a realização de classificação de risco e o tempo porta-antibiótico no paciente com suspeita de sepse.

**Método:**

estudo de coorte retrospectivo, com amostra de 232 pacientes com suspeita de sepse atendidos no pronto atendimento. Foram distribuídos em 2 grupos: com e sem classificação de risco. Identificado o tempo porta-antibiótico, realizou-se análise de variância de um fator com *post hoc* de Bonferroni ou teste T-Student independente para variáveis quantitativas contínuas; testes de correlação de Pearson, correlação bisserial por pontos ou correlação bisserial para análises de associação; e procedimento de *bootstrap* quando não havia distribuição normal de variáveis. Para a análise dos dados foi utilizado o *software Statistical Package for the Social Sciences*.

**Resultados:**

o tempo porta-antibiótico não diferiu entre o grupo que recebeu classificação de risco comparado ao que não foi classificado. O tempo porta-antibiótico foi significativamente menor no grupo que recebeu classificação de risco de alta prioridade.

**Conclusão:**

não houve associação entre o tempo porta-antibiótico e a realização ou não da classificação de risco, tampouco com internação em enfermaria e em unidade de terapia intensiva, ou com o tempo de internação hospitalar. Observou-se que quanto maior a prioridade, menor o tempo porta-antibiótico.

**Highlights:**

**(1)** Performing risk classification has a favorable impact on the management of sepsis.

**(2)** higher the clinical priority ranked, the shorter the door-to-antibiotic time.

**(3)** Door-to-antibiotic time did not differ between classified and unclassified groups.

**(4)** Assertive risk classification is more important than its implementation itself.

## Introduction

The overcrowding of emergency services has shown exponential growth since the 1990s in several countries, and remains a worldwide phenomenon ^(^ 1 ^-^ 2 ^)^. In Brazil, in order to reorganize the care of these services and minimize the risks and damage caused by their overcrowding, it was proposed, through the National Humanization Policy, the reception with risk classification ^(^ 3 ^-^ 4 ^)^. 

Among the existing risk classification protocols, the Manchester Protocol, developed by nurses and doctors in the United Kingdom, stands out. Its strategy is to establish, among the patients presenting to the emergency department (ED), which ones should have priority in care, based on clinical criteria. The methodology of this protocol is based on the main complaint of the patient, which directs the nurse to a clinical condition flowchart. Each flowchart contains discriminators that guide the investigation and, according to the patient’s responses, the severity or clinical risk is classified ^(^ 5 ^)^. 

Currently, one of the main causes of morbidity and mortality worldwide is sepsis. Sepsis is the presence of life-threatening organ dysfunction caused by a dysregulated host response to infection ^(^ 6 ^)^. An extrapolation of data from high-income countries suggests global estimates of 31.5 million cases of sepsis and 19.4 million cases of severe sepsis, with a potential of 5.3 million deaths annually ^(^ 7 ^)^. 

The earlier and more assertive the therapeutic approach, the more possibilities for positive outcomes, with improved survival of patients with sepsis. In patients with a high probability of sepsis or possible septic shock, it is recommended to administer an antimicrobial immediately or within one hour after sepsis is recognized or suspected ^(^ 8 ^)^. Door-to-antibiotic time (DAT) is the time in hours from the arrival of the patient to the ED until the beginning of the administration of the first antibiotic ^(^ 9 ^)^. 

The initial suspicion of sepsis by the Manchester Protocol flowcharts/discriminators is quite sensitive, and will usually determine the opening of the sepsis protocol. On the other hand, attention should be given to patients without clinically apparent organic dysfunction and who can be classified as less urgent clinical priority, which would allow medical attention within 120 minutes, and could lead to non-adherence to the protocol. However, it is not the function of the professional responsible for risk classification to open the sepsis protocol, as this could compromise the overall performance of the triage process and delay the care process of other patients in situations as serious as this disease. Nevertheless, when there is a suspicion of sepsis, the professional can proceed with the protocol by calling the doctor, who will follow it up or not ^(^ 10 ^)^. 

In 2018, the ED of the participating institution implemented the Manchester Risk Classification System in order to ensure that medical attention occurs according to the response time determined by the clinical severity of the patient. In September 2019, the managed sepsis protocol was established in this ED, consisting of a doctor’s office and an Observation and Risk Classification Room, the latter operating from Monday to Saturday from 9:00 am to 9:00 pm. At the time, it was established that sepsis should be suspected in every patient with at least two signs of Systemic Inflammatory Response Syndrome (SIRS), such as: hypothermia (< 35ºC) or hyperthermia (> 37.8ºC), leukocytosis (> 1200 or left shift > 10%) or leukopenia (< 4000), tachycardia (> 90 bpm), and tachypnea (> 20 bpm); and one organ dysfunction criterion: oliguria, hypotension (systolic blood pressure < 90 mmHg), lowered level of consciousness, dyspnea, or desaturation (<92%). It was also determined that for every patient suspected of having sepsis, the Sepsis Monitoring Form would be filled out, in which all information regarding the patient’s initial care would be recorded.

Considering that time is a determining factor for better outcomes in the context of sepsis and risk classification can prioritize patient care, the following question then arises: the presence of risk classification in an ED reduces DAT in patients with suspected sepsis?

The general objective of the study is to evaluate the association between risk classification and DAT in patients with suspected sepsis. The specific objectives include verifying whether there was a difference in time for the administration of antibiotics since the patient’s entry into the ED, according to whether or not the risk classification was performed and according to the color classification (priority), comparing the DAT with the patient’s outcome (admission to the Intensive Care Unit - ICU, admission to the infirmary, discharge or death), and verifying the association between DAT and length of stay.

## Method

### Type of study

This is a retrospective cohort study.

### Place

The study was carried out in a public hospital of medium complexity in Belo Horizonte, Minas Gerais, Brazil. It currently has 82 beds, 10 of which are in the ICU, and provides support to patients from a wide range of clinics.

### Period

The period between September 1, 2019 and September 30, 2021 was considered.

### Population

People assisted in the hospital’s ED between September 1, 2019 and September 30, 2021, to achieve a representative sample.

### Selection criteria

Adult individuals treated at the hospital’s emergency department were included, for whom the suspicion of sepsis was raised and the institution’s specific form, called Sepsis Monitoring Form, was completed.

Patients who had the sepsis protocol initiated, but the diagnosis of infection was later discarded, as well as those individuals referred to exclusive palliative care, regardless of having received initial antibiotic therapy, were excluded.

Individuals whose time of suspected sepsis or antibiotic administration was not recorded were considered losses.

### Sample definition

To define the sample, the critical value for the confidence level of 95% (Z _a/2_ = 1.96) was adopted, with a margin of error of 2.1% and based on a finite population of 260 individuals assisted in the same period in hospital ED. 

The study group consisted of patients submitted to Risk Classification, and patients not submitted to it as a control group. The risk-classified group was made up of patients admitted to the ED from Monday to Saturday between 9:00 am and 9:00 pm, and the control group comprised those admitted to the ED on Sundays and on other weekdays between 9:00 pm and 9:00 am.

### Study variables

DAT was considered as the primary outcome. As secondary outcomes, the following variables were included: ICU admission, infirmary admission, discharge, death and length of stay. The following variables were also analyzed: sex, age, Risk Classification, Risk Classification color, focus of infection and antibiotic used.

### Data collection

Data collection was carried out from different sources. Firstly, the sepsis monitoring forms were tracked, which included the following information: the patient’s age and gender, whether there was a Risk Classification, the color of the Risk Classification (reflects the priority of care), the focus of suspected infection, the antibiotic administration starting time and the antibiotic administered. This form is filled out for all patients receiving care in the ED as soon as the suspicion of sepsis is raised. The two-year period for the search of individuals (September 2019 to September 2021) is due to the fact that this form was inserted concomitantly with the implementation of the Managed Sepsis Protocol in September 2019. All patients had DAT calculated for comparison between study and control groups. The DAT is the one between the patient’s entry into the ED (when the care form is opened at the counter) and the start of the administration of the antibiotic. The patient’s entry time into the ED was identified in the *Sistema Integrado de Gestão de Assistência à Saúde* (SIGAS) of the *Instituto de Previdência dos Servidores Militares de Minas Gerais* (IPSM). The antibiotic administration time was identified in the sepsis monitoring form. 

The information about whether the patient underwent Risk Classification, as well as the color of the Classification, were confirmed directly in the care record filed at the *Serviço de Arquivo Médico e Estatística* (SAME). 

Information related to the patient’s clinical outcome (admission to the ICU or the infirmary, hospital discharge or death) was collected from the patient’s medical record through the *Sistema Integrado de Gestão à Saúde* (SIGS). 

All collected information was entered into a database organized in a spreadsheet and exported to the statistical package for analysis.

### Data treatment and analysis

Continuous quantitative variables of study outcome were compared via one-way analysis of variance (one-way ANOVA) with Bonferroni *post hoc* for multiple comparisons or independent Student’s t-test when comparisons were performed only between two groups. Association analyzes were performed using Pearson correlation tests, point-biserial correlation or biserial correlation, according to the types of variables: continuous or discrete/categorical, respectively. As some variables did not present a normal distribution, assessed by the Shapiro-Wilk test, the bootstrap procedure was used with 500 replicates of simple sampling. This is a robust and reliable method to provide valid confidence intervals when normal distribution of residuals is not observed and/or the sample is small ^(^ 11 ^)^. 

The sociodemographic and clinical characteristics of the sample were expressed as mean (minimum - maximum) or absolute and relative frequency. The results of the comparison analyzes were expressed as mean ± standard deviation, confidence interval of differences between means and p-value. The significance level adopted was alpha less than 5%.

Data were analyzed using the Statistical Package for Social Sciences – SPSS software (version 20.0).

### Ethical aspects

This study was developed in accordance with the provisions of Resolutions nº 466/2012 and 510/2016 of the National Health Council, which establish guidelines and standards for research involving human beings ^(^ 12 ^-^ 13 ^)^. It was submitted to the analysis and approval of the Research Ethics Committee of the Institution of the study and the *Comissão Nacional de* Ética *em Pesquisa* (CONEP) – *Certificado de Apresentação de Apreciação* Ética (CAAE) nº 57746822.8.0000.5155. 

As the data were collected retrospectively, the Free and Informed Consent Form was waived. However, the Term of Commitment to Use Data (TCUD) was carried out, since the research involved the use and collection of information in the Institution’s database and patient records.

The risk to the research individuals involved the eventual exposure of information related to their data contained in medical records and attendance sheets. The team of researchers is committed to minimizing this risk, disclosing results only at scientific events and publication in indexed journals. Also, to preserve the privacy of individuals, the anonymity of the collected data was maintained.

## Results

**Figure 1 -:**
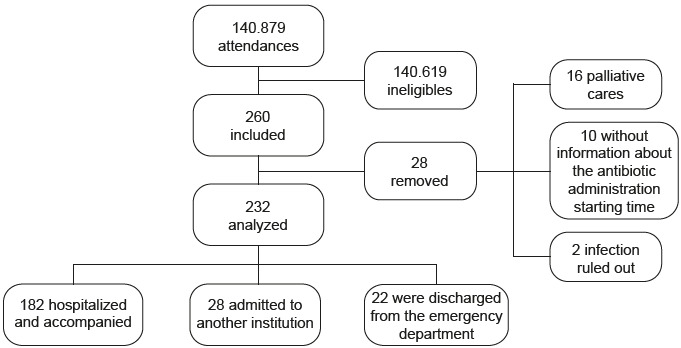
Flowchart of the sample composition of this study. Belo Horizonte, MG, Brazil, 2022

During the studied period, 140,879 consultations were performed in the hospital’s ED, of which 28,026 (19.9%) underwent risk classification. A total of 260 patients eligible for the study with suspected sepsis were identified. Twenty-eight patients were removed (16 patients in palliative care, 10 without information about the antibiotic administration starting time, and 2 whose infection was ruled out). Therefore, 232 patients were analyzed ( Figure 1). After hospitalization, 182 patients were followed up. 

Table 1 contains the sociodemographic and clinical characteristics of the sample. 

**Table 1 -:** Sociodemographic and clinical characteristics of patients suspected of sepsis (n*= 232). Belo Horizonte, MG, Brazil, 2022

Variables	Descriptive statistics
**Age**	68.2 (15.0 – 101.0)
**Sex (n / %)** ^†^		
Female	115 / 49.6
Male	117 / 50.4
**Risk Classif.** ^‡^ **(n / %)** ^†^		
Yes	97 / 41.8
No	135 / 58.2
**Risk Classif.** ^‡^ **color (n / %)** ^†^		
Blue	5 / 2.2
Green	16 / 6.9
Yellow	37 / 15.9
Orange	38 / 16.4
Red	1 / 0.4
Unclassified	135 / 58.2
**Hospitalization (n / %)** ^†^		
ICU ^§^	105 / 45.2
Infirmary	77 / 33.2
Did not hospitalize	50 / 21.6
**Hospital discharge alive (n / %)** ^†^		
Yes	147 / 63.3
No	35 / 15.1
Unaccompanied patients	50 / 21.6

^*^n = Sample; ^†^n / % = Absolute Frequency / Relative Frequency; ^‡^Classif. = Classification; ^§^ICU = Intensive Care Unit. Data expressed as: mean (minimum – maximum) or absolute / relative frequency

The sites of infection identified as probable in patients with suspected sepsis were: pulmonary (33.2%), urinary (32.3%), abdominal (9.1%), undefined (3.9%), other (17.6%), and not informed (3.9%). The antibiotics used in therapy were: ceftriaxone (29.7%), ceftriaxone with azithromycin (13.8%), piperacillin with tazobactam (7.8%), meropenem (7.3%), ceftriaxone with metronidazole (6%), amoxicillin with clavulanate (5.6%), cefepime (4.3%), other (19.5%), not informed (6%).

DAT did not differ between the group that received Risk Classification and the group that was not classified: 133.2 ± 94.8 vs 152.8 ± 108.0 (95% CI: -9.4 to 39.1; p= 0.175) and 67.6 ± 48.3 vs 87.0 ± 118.8 (95% CI: -2.8 to 21.4; p= 0.118), respectively.

The DAT mean was significantly lower (p<0.01) in the group that received a high priority Risk Classification (with orange or red color), compared to intermediate priority groups (classified as yellow), low priority (classified as green or blue), and those who did not receive a risk classification, as shown in Table 2. 

Table 3 shows only the patients admitted to the reference hospital and followed up (n=182). DAT was not different between infirmary and ICU admission groups. The same was observed in relation to the groups that were discharged alive or not (p>0.05 for all comparisons). 

**Table 2 -:** Comparison of DAT ^*^ between priority classifications for care / colors among sepsis suspects (n ^†^= 232). Belo Horizonte, MG, Brazil, 2022

Priority classification for care/colors	DAT ^*^(min) ^§^ Mean ±SD ^||^	Mean of differences (95% CI ^‡^)			
		No risk classification	Low priority (Blue/Green)	Intermediate priority (Yellow)	High priority (Orange/Red)
No risk classification	157.3 ± 8.6 ^¶^	-------	-35.5 (-99.9 to 18.1)	15.8 (-19.0 to 47.3)	60.1 (36.3 to 83.0)
Low priority (Blue/Green)	192.8 ± 21.7 ^¶^	35.5 (-18.1 to 99.9)	-------	51.3 (-6.4 to 114.1)	95.6 (42.3 to 155.8)
Intermediate priority (Yellow)	141.4 ± 16.4 ^¶^	-15.8 (-47.3 to 19.0)	-51.3 (-114.1 to 6.4)	-------	44.3 (13.6 to 75.2)
High priority (Orange/Red)	97.2 ± 15.9	-60.1 (-83.0 to -36.3)	-95.6 (-155.8 to -42.3)	-44.3 (-75.2 to -13.6)	

^*^DAT = Door-to-Antibiotic Time; ^†^n = Sample; ^‡^95% CI = 95% Confidence Interval; ^§^min = minutes; ^||^SD = Standard Deviation; ^¶^p (significance level) <0.01 compared to high priority care group (Orange/Red). Data expressed as: mean ± standard deviation; mean of differences (95% confidence interval of comparisons)

**Table 3 -:** Mean values and 95% CI ^*^ of associations between DAT ^†^ and hospitalization (infirmary and ICU ^‡^) and hospital discharge (n§= 182). Belo Horizonte, MG, Brazil, 2022

Variables	DAT ^†^ (min ^)||^ mean ± standard deviation [95% CI* comparisons] (p-value ^¶^)	
Hospitaliz ^**^ Infirmary ICU ^‡^	146.05 ± 109.65 152.66 ± 101.15 [-37.72 to 26.22] (0.686)
Hospital discharge alive Yes No	148.39 ± 109.86 154.23 ± 84.92 [-25.97 to 39.09] (0.732)

*95% CI = 95% Confidence Interval; ^†^DAT = Door-to-Antibiotic Time; ^‡^ICU = Intensive Care Unit; §n = Sample; ^||^min = Minutes; ^¶^ p-value = p value (significance level); **Hospitaliz. = Hospitalization. Data expressed as: mean ± standard deviation; [95% confidence interval of comparisons between groups (p value)]

There was no association between DAT and whether or not the Risk Classification was performed (r _bp_= -0.09; 95% CI: -0.22 to 0.04; p=0.177) or with the length of hospital stay (r= -0.06; 95% CI: -0.13 to 0.11; p=0.430). However, when analyzing Risk Classification by severity/color with DAT, a low magnitude, inverse and statistically significant association was observed (r _b_= -0.14; 95% CI: -0.26 to -0.01; p=0.045), showing that the greater the severity, the lower the DAT. 

## Discussion

Most of the study population was composed of elderly people, with a mean age of 68.2 years, and a slight majority of men (50.4%). These findings corroborate those of other studies ^(^ 14 ^-^ 15 ^)^. The fact that individuals over 60 years old are more affected is explained by the presence of chronic diseases, comorbidities, frailty and functional impairment (characteristic immunosenescence of the elderly). Added to current changes in demographic characteristics are population aging and increased life expectancy ^(^ 16 ^)^. 

Most patients in this study (58.2%) were not submitted to Risk Classification. This data can be justified by the fact that most of the data collection period occurred during the COVID-19 pandemic (Coronavirus - 2019). During this period, it was necessary to divide the access to the ED into two entrances: one to care for patients with suspected COVID-19, and another for other conditions. The risk classification service remained only for patients who did not show flu signs, without suspicion of COVID-19. Individuals suspected of having COVID-19 represented a significant percentage of the consultations generated in the hospital’s ED during the study period.

Among patients who underwent Risk Classification, most were classified as orange (16.4%) or yellow (15.9%). A systematic review found that the sensitivity of high-priority detection for critical illness outcomes varied and was, on average, lower for severe sepsis (36 to 74%), which reveals a challenge for Risk Classification nurses. A total of 20 studies captured sensitivity and specificity data for designations of triage levels for the outcome of hospital admission. The sensitivity of assigning inpatients highest priority (levels 1 to 3 = red, orange and yellow in the Manchester system) was relatively high, with only 3 of 20 studies reporting less than 70%. However, all studies reported hospital admissions occurring for low-priority patients (levels 4 and 5 = green and blue in the Manchester system ^(^ 17 ^)^. 

The main suspected sites of infection were pulmonary (33.2%) and urinary (32.3%), similar to results found in other studies. One of them demonstrated that almost half of the cases of sepsis were secondary to pulmonary infections (49%) ^(^ 18 ^)^. The predominance of the pulmonary focus in patients with sepsis may reflect the fact that the majority of the population under analysis was composed of elderly people, and this group has a higher rate of involvement with chronic diseases and is usually more predisposed to respiratory infections. Urinary and abdominal foci usually alternate as second and third most frequent sites of infection ^(^ 19 ^)^. 

The use of the antibiotic ceftriaxone in the initial treatment of septic conditions stood out. It was applied alone in 29.7% of cases, and associated with azithromycin and metronidazole in 13.8% and 6.0% of cases, respectively.

The early use of antimicrobials can improve the prognosis of patients with sepsis, which is why international guidelines recommend the empirical use of broad-spectrum antibiotics, although they warn against their excessive use, which is commonly associated with the development of bacterial resistance ^(^ 20 ^)^. Therefore, the Latin American recommendation emphasizes that combined therapy should be reserved for situations in which data from the local microbiota or the clinical situation suggest a greater probability of infection by resistant germs ^(^ 21 ^)^. In addition, it recommends that empirical antibiotic therapy be discontinued as soon as the pathogen and its antimicrobial sensitivity profile are identified, as well as significant clinical improvement, reinforcing the importance of continuous reassessment and focus control. 

Among the patients included in the study, 78.4% were admitted to the participating hospital. These individuals were analyzed after hospital admission, identifying 57.7% and 42.3% of them admitted to the ICU and the Infirmary, respectively. Regarding the outcome of hospitalizations, 80.7% of patients were discharged alive and 19.3% died. Other patients could not be followed up during their hospital stay, with unknown hospital outcomes, as they were admitted to other institutions, which represented 21.6% of the sample initially analyzed in the ED. It is noteworthy that the sample consisted of patients with suspected sepsis. A multicenter study carried out in Brazil showed that one third of intensive care beds are occupied by septic patients, with a mortality rate of 55.7% ^(^ 22 ^)^. 

A study on the trend analysis of mortality from sepsis in Brazil and by regions from 2010 to 2019 pointed out that, when compared to other countries, both developed and underdeveloped, deaths from sepsis in Brazil are in a global trend of high prevalence, with a mortality rate of 22.8 deaths per 100 thousand inhabitants ^(^ 16 ^)^. 

Just over a fifth of the study patients (21.6%) were not admitted to the participating hospital. Therefore, their circumstances, clinical situations and discharge conditions were not analyzed in this study. It should be noted that the population was composed of patients with suspected sepsis. It is believed that, for the portion that was discharged from the ED without indication of hospitalization (19.2% of the initial sample), sepsis was ruled out and/or they were stable patients, with indication of home treatment and outpatient follow-up. There is a study that points out that ED discharges of patients with clinical definition of sepsis reflect misdiagnosis, inadequate treatment judgments, patient preferences or adequate triage of low-risk patients for outpatient treatment ^(^ 23 ^)^. 

The mean DAT was lower among patients submitted to risk classification when compared to those not classified, as specified in Table 2, but without statistical significance. International sepsis management guidelines recommend, for adults with possible septic shock or high likelihood of sepsis, administration of antimicrobials immediately, ideally within 1 hour of recognition, and within 3 hours for adult patients with possible sepsis without shock ^(^ 8 ^)^. 

In a study of 10,811 eligible patients, the median DAT was 166 min (interquartile range, 115-230 min) and the 1-year case fatality was 19%. After adjustment, each additional hour from arrival at the ED to initiation of antibiotics was associated with a 10% increase (95% confidence interval [95% CI] 5-14; P < 0.001) in 1-year case fatality ^(^ 24 ^)^. In contrast, a survey carried out in an Emergency Department with patients with septic shock did not find an association between in-hospital lethality and the time from emergency triage to administration of antibiotics during the first 6 hours of resuscitation ^(^ 25 ^)^. 

In this study, DAT was significantly lower in the group that received a “high priority” risk classification compared to the others, establishing an inverse relationship, that is, the higher the priority, the lower the DAT. This result points to the importance of an assertive risk classification. Such procedure aims at the early identification of the patient who presents the possibility of clinical worsening and establishes priority for the care of the most serious conditions. The updated version of the Manchester Triage System, more recent than the one used in the hospital at the time of the research, presents the discriminator “possible sepsis” in 40 flowcharts. This discriminator indicates the assessment of pulse, temperature, change in level of consciousness, respiratory rate and systolic blood pressure. The parameters are established in the protocol and, upon some alteration, direct the classifier to select the mentioned discriminator. Established criteria assign high sensitivity for early detection of sepsis and determine a very urgent clinical priority (orange).

It should be noted that, in this study, patients classified as orange had some critical condition that signaled to the classifier the clinical priority of very urgent. However, the classification protocol used at that time did not yet include the “possible sepsis” discriminator. It is believed that this update allows the classifier to direct, in a more agile way, such conditions so that the care team triggers the care sequence that involves investigation, diagnosis and early initiation of treatment.

Other studies also showed that the time for antibiotic administration was shorter in patients classified as having higher priority, when compared to the others ^(^ 26 ^-^ 27 ^)^. Furthermore, patient classification for lower priorities of care has been shown to be an independent risk factor for delay in administering the first dose of antibiotic ^(^ 28 ^)^. 

DAT was not different between patients admitted to the ICU or the infirmary, nor was it associated with hospital survival. However, several epidemiological particularities that may influence the patient’s outcome were not taken into account for this analysis, such as, for example, pre-existing comorbidities. The assertiveness in the diagnosis of sepsis and septic shock was also not evaluated, as well as the use of antimicrobials, which can directly influence the assessment of mortality. In addition, it is known that the length of stay of patients with sepsis is a factor of great influence on their prognosis, since the patient’s stay, especially in the ICU, requires a greater number of procedures and intensive care, which can give rise to other infectious foci ^(^ 29 ^)^. 

Another relevant factor in the study results is the implementation of the managed protocol as a strategy that aligns the best evidence in sepsis treatment with organized actions for recognition and treatment, team education, composition of teams of professionals to support actions, as well as measurement of indicators to evaluate results ^(^ 30 ^)^. One study showed that this strategy increased by eight times the chances of the patient receiving treatment in one hour, in addition to reducing mortality by 10.33% ^(^ 31 ^)^. 

Other than DAT, short-term outcomes have not been studied. Confounding effect may increase with length of hospital stay. The risk classification, according to the routine of the study site, was interrupted from 9 pm to 9 am from Monday to Saturday, and did not occur on Sundays, which may have influenced the fact that no statistical difference was found in DAT between patients submitted or not to risk classification. This fact represents one of the limitations of this study, since the times when the risk classification was not available, namely weekends and night periods, are traditionally periods with less supply of resources, but also with less flow of patients. As it was not possible to determine the waiting time in the risk classification, it was also not possible to infer its influence on the results, such as, for example, delays in conduct.

Another limitation of the study is its retrospective and observational design, so that individuals were not allocated to the study or control groups according to specific criteria, but only due to the conditions imposed by the availability of human resources at specific times and the strong impact resulting from the COVID-19 pandemic.

The study was conducted at a single center and with a relatively small participant size. The retrospective nature of the study and possible poor documentation led to the lack of some information, which made patient selection difficult. Another limitation was the fact that the inclusion period coincided with the COVID-19 pandemic. At this time, the risk classification was directed to only patients without flu symptoms. In this way, there was a significant reduction in the number of classified patients that reflected in the structure of the study, in which the majority of the population was not subjected to risk classification, but also allowed the composition of a significant control group.

## Conclusion

This study showed that DAT did not differ between the group that received risk classification when compared to the group that was not classified. On the other hand, it was noticed that there is an association between risk classification by priority/color and DAT. That is, the higher the clinical priority received in the risk classification, the lower the DAT. High priority for care according to the Manchester Risk Classification Protocol was also significantly associated with a lower DAT when compared to unclassified patients. There was no association between DAT and hospitalization in the ICU or the Infirmary, nor with hospital survival. There was also no association between DAT and length of hospital stay.

The results of this study allow us to assess the risk classification in the ED as relevant and its favorable impact on the management of patients with sepsis. They also allow highlighting how important it is to expand discussions and studies about risk classification processes, since assertive classification with determination of adequate clinical priority contributes greatly to conducting clinical management in a timely manner. However, it is evident that carrying out an assertive classification is much more important than its implementation itself.
